# Design of a Network Permutation Entropy and Its Applications for Chaotic Time Series and EEG Signals

**DOI:** 10.3390/e21090849

**Published:** 2019-08-30

**Authors:** Bo Yan, Shaobo He, Kehui Sun

**Affiliations:** 1 College of Computer and Electrical Engineering, Hunan University of Arts and Science, Changde 415000, China; 2 School of Physics and Electronics, Central South University, Changsha 410083, China

**Keywords:** permutation entropy, complexity, network, chaotic system, EEG signal

## Abstract

Measuring the complexity of time series provides an important indicator for characteristic analysis of nonlinear systems. The permutation entropy (PE) is widely used, but it still needs to be modified. In this paper, the PE algorithm is improved by introducing the concept of the network, and the network PE (NPE) is proposed. The connections are established based on both the patterns and weights of the reconstructed vectors. The complexity of different chaotic systems is analyzed. As with the PE algorithm, the NPE algorithm-based analysis results are also reliable for chaotic systems. Finally, the NPE is applied to estimate the complexity of EEG signals of normal healthy persons and epileptic patients. It is shown that the normal healthy persons have the largest NPE values, while the EEG signals of epileptic patients are lower during both seizure-free intervals and seizure activity. Hence, NPE could be used as an alternative to PE for the nonlinear characteristics of chaotic systems and EEG signal-based physiological and biomedical analysis.

## 1. Introduction

Measuring the nature of the complexity of obtained time series can provide a better understanding of nonlinear systems. This has aroused much interest from researches. Currently, the complexity of different kinds of nonlinear time series such as EEG signals [1,2], ECG signals [3,4], EMG signals [5], stock data [6], traffic time series [7], and tea category identification [8] has been investigated. Meanwhile, the complexity analysis of chaotic systems has been reported in many literature works, and the dynamics of chaotic systems has garnered further study [9,10,11,12]. It also provides a reference for the parameter choice of chaotic systems in real applications [13].

To measure the complexity of nonlinear time series, various types of algorithms are designed. Firstly, according to whether the Shannon entropy [14] is used or not, the complexity measuring algorithms can be categorized into the complexity methods and entropy methods. The Kolmogorov complexity algorithm [15,16], C${}_{0}$ algorithm [17], ApEn [18], SampEn [19], FuzzyEn [20], the Lempel–Ziv complexity measuring algorithm [21], and the statistical complexity measure method [22] belong to the complexity methods, while permutation entropy (PE) [23], spectral entropy (SE)[24], and wavelet entropy (WE) [25] belong to the entropy algorithms. Meanwhile, according to which domain is used to measure the complexity, the complexity measuring algorithms can be defined as time domain method and frequency domain methods. The SE algorithm [24], C${}_{0}$ algorithm [17], and WE algorithm [25] are frequency domain methods since the Fourier transformation or the wavelet transformation is used. However, most of the other algorithms measure complexity directly in the time domain. Recently, Coast et al. [26] proposed the multi-scale coarse graining process and designed the multiscale entropy algorithm; thus, complexity can be analyzed in a multiple scale sense. After this, many different multiscale complexity algorithms such as the multiscale PE (MPE) algorithm [27], the multiscale SampEn algorithm [28], the multiscale permutation Rényi entropy [29], and the multivariate multiscale entropy [30] were designed. Among those methods, the PE algorithm and MPE algorithm have a fast estimation speed and are gaining more and more academic attention. Therefore, this article will concentrate on the PE algorithm.

In 2002, Bandt and Pompe [23] proposed the PE algorithm based on the patterns deduced from constructed vectors. However, it was indicated in many literature works that the PE algorithm has some drawbacks that cannot always measure complexity effectively. For instance, Zunino et al. [31] figured out that the Bandt–Pompe method for processing equal values could lead to erroneous conclusions. Until now, how to improve the PE algorithm is still an open project. At present, several improved PE algorithms have been proposed. Bian et al. [32] modified the PE algorithm by mapping the equal value onto the same symbol (rank), and the modified PE (mPE) algorithm was designed. Fadlallah et al. [33] proposed the weighted PE (WPE) algorithm, and EEG signals were analyzed to verify its effectiveness. Azami et al. [34] indicated that PE does not consider the average of the amplitude values and equal amplitude values and designed the amplitude-aware permutation entropy (AAPE). Chen et al. [35] proposed the improved PE (IPE) algorithm by introducing a symbolic process and combining some advantages of previous modifications of PE. As a result, IPE has more patterns and better robustness for noise-polluted time series. Those algorithms have better measuring results when comparing with the original PE algorithm. However, they do not consider the relationship between different patterns since they actually treat the vectors as independent units. Currently, approaches for complexity analysis of nonlinear time series using networks is a hot topic [36,37,38,39,40]. It could provide many possibilities for further investigation of the complexity algorithms. Specifically, we will introduce the connections between different vectors and build the network of the time series, where the Bandt–Pompe patterns and their weights are used.

At present, there are many linear and nonlinear method-based analyses for EEG signal processing applications. Gao et al. [41] developed an EEG-based spatial-temporal convolutional neural network for driver fatigue evaluation, and it allows further implementations in brain–computer interface online systems. Chai et al. [42] introduced the independent component by the entropy rate bound minimization analysis (ERBM-ICA) for the feature extraction of the EEG signals where the Bayesian neural network is used for the classification. Meanwhile, complexity [43], dynamically identifying relevant EEG channels [44], and EEG fractal and spectral analysis [45] have aroused the interest of researchers. Moreover, removal of movement artifacts, ICA theory, and signal processing methods for EEG signals are recent hot topics [46,47,48,49,50,51]. These studies provided a solid foundation for the EEG signal applications and have resulted in a great amount of significant and challenging subjects in life sciences. As mentioned above, complexity measure algorithms can provide powerful analytical tools for the analysis of EEG signals. In this paper, the complexity of the EEG signals [52] is analyzed using the designed network PE algorithm.

The rest of the paper is organized as follows. In Section 2, the PE, WPE, and IPE algorithms are presented; the problems are discussed, and the network PE (NPE) algorithm is proposed. In Section 3, the proposed NPE is applied to analyze the complexity of different kinds of chaotic systems. In Section 4, the NPE algorithm is used to measure the complexity of the EEG signals of epileptic patients. Finally, the results are summarized.

## 2. Improving the Permutation Entropy

### 2.1. The Original Permutation Entropy

To obtain the probability distribution, the first step is to define the patterns. The Bandt–Pompe pattern-based [23] probability distribution is given as follows.

Step 1: For a given time series {x(n),n=1,2,3,⋯,N} and reconstruction dimension *d*, the reconstructed series is denoted by:

(1) X(i)={x(i),x(i+1),⋯,x(i+d−1)},

where i=1,2,⋯,N−d+1.

Step 2: The vector X(i) can be arranged in increasing order $\pi =(r_{0},r_{1},\cdots ,r_{d-1})$, where:

(2) $$x_{i+r_{0}}\leq x_{i+r_{1}}\leq \cdots \leq x_{i+r_{d-1}}.$$

Obviously, there are d! possible order patterns.

Step 3: Define the order pattern of X(i) as $\pi_{j}$. If we give each pattern $\pi_{j}$ a value, a pattern series {s(i),i=1,2,⋯,N−d+1} can be obtained. The Bandt–Pompe probability distribution $p(\pi_{j})$ is denoted by:

(3) $$p\left(\pi_{j}\right)=\frac{\#\left\{s\left(i\right)\left|i\leq N-d+1;s\left(i\right)=\pi_{j}\right.\right\}}{N-d+1}.$$

**Figure 1:**
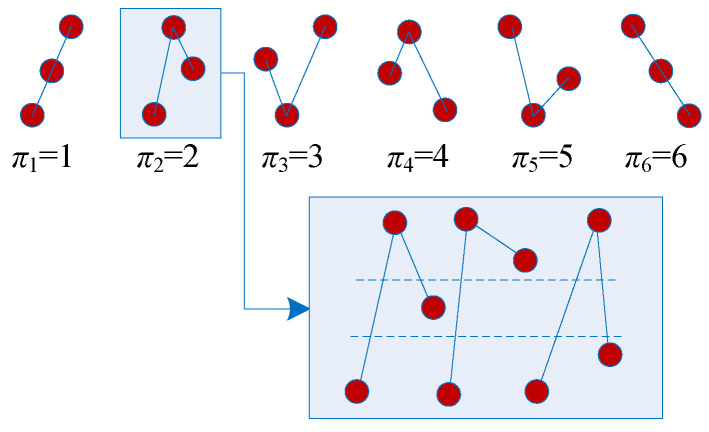
Bandt and Pompe patterns with d=3.

Let us take d=3 as an example; there are six possible patterns: $\left\{\pi_{1},x_{1}\leq x_{2}\leq x_{3}\right\}$, $\left\{\pi_{2},x_{1}\leq x_{3}\leq x_{2}\right\}$, $\left\{\pi_{3},x_{2}\leq x_{1}\leq x_{3}\right\}$, $\left\{\pi_{4},x_{3}\leq x_{1}\leq x_{2}\right\}$, $\left\{\pi_{5},x_{2}\leq x_{3}\leq x_{1}\right\}$, and $\left\{\pi_{6},x_{3}\leq x_{2}\leq x_{1}\right\}$. The six different patterns can also be found in Figure 1. In our implementation, we used the MATLAB function [Reorder,index]=sort(·) for each vector. The pseudocode for obtaining the pattern is shown in Algorithm 1. For each pattern, there is a unique return value. For instance, in the pattern $\left\{\pi_{2},x_{1}\leq x_{3}\leq x_{2}\right\}$, $index=\left[r_{0},r_{1},r_{2}\right]=\left[1,3,2\right]$. Its value is calculated by $S=r_{0}\times 10^{2}+r_{1}\times 10^{1}+r_{2}\times 10^{0}=132$. As a result, different patterns have different values.

**Algorithm 1** Set a unique value for a given pattern (vector *X*); the function name is GetPattern(·).
**Input:** *X*, *d***Output:** 
*S*
 
g=zeros(1,d);
 
**for** i=1→d **do**
  
$g\left(i\right)=10^{d-i};$
 
**end for**
 [temp,index]=sort(X); $S=g*index^{T}$;


According to the definition of probability distribution *p* associated with the time series {x(i):i=1,2,⋯,N}, the permutation entropy (PE) algorithm [23] is defined as:

(4) $$\mathrm{PE}\left(x^{N},d\right)=-\frac{1}{\ln(d!)}\sum_{j=1}^{d!}p_{j}\left(\pi \right)\ln\left(p_{j}\left(\pi \right)\right).$$

The normalized entropy is evaluated for this “permutation” probability distribution. Usually, the range of the embedded dimension *d* is {3,4,⋯,7} [23].

### 2.2. The Deficiency of the Bandt–Pompe Probability Distribution

Firstly, the existing problems of the PE algorithm are listed as follows:

- As shown in Figure 1, there are many different cases for each pattern, but the Bandt–Pompe patterns are recognized as the same case. Thus, the Bandt–Pompe patterns cannot detect the nonlinearity in time series effectively.
- The pattern series {s(n),n=1,2,⋯,N} of a periodic time series is also periodic [53]. The corresponding Bandt–Pompe probability distribution $p(\pi_{j})$ could be a uniform distribution or other cases. Thus, sometimes, PE measuring results cannot distinguish the periodic state and the chaotic state [53].

As for the sensibility of the Bandt–Pompe patterns, some explanation are presented here. The vectors such as [1,2,3], [1.1,1.2,3], and [1,2.8,3] will be symbolized as the same pattern $\left\{\pi_{1},x_{1}\leq x_{2}\leq x_{3}\right\}$. Chen et al. [35] proposed the improved permutation entropy (IPE), where the probability distribution is obtained as follows.

Step 1: The quantization process is given by:

(5) $$S\left(x\right)=\left\{\begin{array}{c} 0,x_{\min}\leq x<\Delta \\ 1,\Delta \leq x<2\Delta \\ \vdots ,\;\;\vdots \\ L-1,\left(L-1\right)\Delta \leq x\leq x_{\max} \end{array}\right.,$$

where $x_{\max}$ and $x_{\min}$ are the maximum and minimum values of the time series *x*. Thus, an integer number ranging from 0–L−1 can be obtained.

Step 2: Let S(:,1) denote the symbolization result of the reconstructed series X(:,1). Then, for the kth column of embedding vectors [35]:

(6) $$S\left(j,k\right)=S\left(j,1\right)+\left\lfloor \frac{X\left(j,k\right)-X\left(j,1\right)}{\Delta }\right\rfloor ,$$

where j=1,2,⋯,N−d+1, 2≤k≤d. As a result, each vector is changed to a symbol vector.

Step 3: For each vector, there are $L^{d}$ possibilities. Thus, the probability distribution is defined as:

(7) $$p_{I}\left({\tilde{\pi }}_{j}\right)=\frac{\#\left\{S\left(i,:\right)\left|i\leq N-d+1;S\left(i,:\right)={\tilde{\pi }}_{j}\right.\right\}}{N-d+1},$$

where $j=1,2,\cdots ,L^{d}$.

Let us give some examples to explain this method. Suppose that $x_{min}=0$, $x_{max}=3$, and L=4; the vectors are given as [1,2,3], [1.1,1.2,3], and [1,2.8,3]. In this case, the patterns are symbolized as 113, 103, and 133. Obviously, they are not the same patterns. In fact, $L^{d}\gg d!$ when *L* takes values larger than *d*. This means more permutation patterns will be obtained. As a result, IPE patterns are more sensitive to the changes in the vectors.

Meanwhile, Fadlallah et al. [33] proposed the weighted PE (WPE) for better measuring results. The WPE is calculated as follows. For the vector X(i), its weight is calculated by:

(8) $$w_{i}=\frac{1}{d}\sum_{k=1}^{d}{\left[x\left(i+k-1\right)-\bar{X}\left(i\right)\right]}^{2}$$

where $\bar{X}\left(i\right)$ is the mean value of vector X(i). The probability distribution is calculated by [33]:    

(9) $$p_{w}\left(\pi_{i}\right)=\frac{\sum_{j=1}^{N-d+1}1_{\left(s_{j}=\pi_{i}\right)}w_{j}}{\sum_{j=1}^{N-d+1}1_{\left(s_{j}\right)}w_{j}}.$$

In this method, the denominator is the summation of weights of all vectors, while the numerator is the summation of weights of each pattern. Compared with PE, WPE considers the weights of the vector; thus, it can provide more information about the nonlinearity of the time series.

Obviously, compared with the original Bandt–Pompe patterns, there is more information considered in the modified methods. The probability distribution is more reliable, and more satisfying measuring results can be obtained for the nonlinear time series.

However, these two methods still cannot solve the second problem illustrated. In our previous work, we pointed out that the PE algorithm cannot always detect the periodic state effectively [53]. Here, we explain the given example again. Suppose that there is a periodic time series, which is given by {1, 2, 3, 4, 5, 1, 2, 3, 4, 5, 1, 2, ⋯}, and the Bandt–Pompe patterns are applied to detect the complexity. When d=3, the Bandt–Pompe probability distribution *P* = [0.6, 0.2, 0.2]. When *d* = 4, the probability distribution *P* = [0.4, 0.2, 0.2, 0.2]. When d≥5, the probability distribution *P* = [0.2, 0.2, 0.2, 0.2, 0.2]. Obviously, an unsatisfying complexity estimation is obtained when d≥5. For WPE and IPE, the pattern series is also periodic, and the deduced probability distribution may be quite balanced, while high complexity measuring results are obtained. The main reason for those improved PE algorithms is that they mainly focused on the patterns, but did not try to establish connections between the patterns. In real applications, we hope that the complexity measuring results are low for those periodic time series, while high for those nonlinear and complex time series. Thus, it is still necessary to modify the PE algorithm.

### 2.3. Network Permutation Entropy

To improve the PE algorithm with a better ability to detect complexity in the nonlinear time series and to identify the periodic time series, the network permutation entropy (NPE) is designed. The key is to use the concept of the “connection” of different vectors. This means that we need to use the knowledge of the network.

For a given time series {x(n),n=1,2,3,⋯,N}, the steps to calculate the NPE are listed as follows.

Step 1: Reconstruct the time series based on Equation (1), then a pattern time series {s(i),i=1,2,⋯,N−d+1} is obtained based on the Bandt–Pompe patterns.

Step 2: Calculate the weights of each vector using Equation (8), and the results are saved in the weight series {w(i),i=1,2,⋯,N−d+1}. In this step, we also calculate another index to decide whether the weights are positive or negative. The index is give by:

(10) $$flag_{i}=mean\left(X\left(i,2:d-1\right)\right)-mean\left(X\left(i,:\right)\right).$$

If $flag_{i}\geq 0$, then the weight w(i) is positive; otherwise, w(i) is negative.

Step 3: For the ith (i≥2) vector X(i,:), we check the vector X(j,:) (j=1,2,⋯,i−1). Suppose that the first vector (X(n,:), n∈[1,⋯,i−1]), which has the same pattern as vector X(i,:), namely, s(i)=s(n), the two vectors are connected if $\left|w\left(i\right)-w\left(n\right)\right|\leq error$. Then, this round of search stops; then, increase the value of *i* by one, and start the next round until i=N−d+1.

Step 4: Record the connected information. Here, we use two variables. One is used to record the probability distribution, and it is denoted as {P(i),i=1,2,⋯,N−d+1} and initialized as tiny constants. In this study, $P\left(i\right)=10^{-5}$. The other one is to record the connection information, which is given by {M(i,j),i,j=1,2,⋯,N−d+1}. The matrix *M* is initialized as a zero matrix. In each step, if two connected vectors X(i,:) and X(n,:) are found, then:

(11) $$\left\{\begin{array}{c} P\left(n\right)=P\left(n\right)+1\\ M\left(i,n\right)=1\\ M\left(n,i\right)=1 \end{array}\right..$$

Step 5: Let:

(12) $$P\left(i\right)=\frac{P\left(i\right)}{\sum_{i=1}^{N-d+1}P\left(i\right)},$$

where i=1,2,⋯,N−d+1. Then, the NPE is defined as:

(13) $$NPE\left(x^{N},d,error\right)=-\frac{1}{\log\left(N-d+1\right)}\sum_{i=1}^{N-d+1}P\left(i\right)\log\left(P\left(i\right)\right).$$

**Figure 2:**
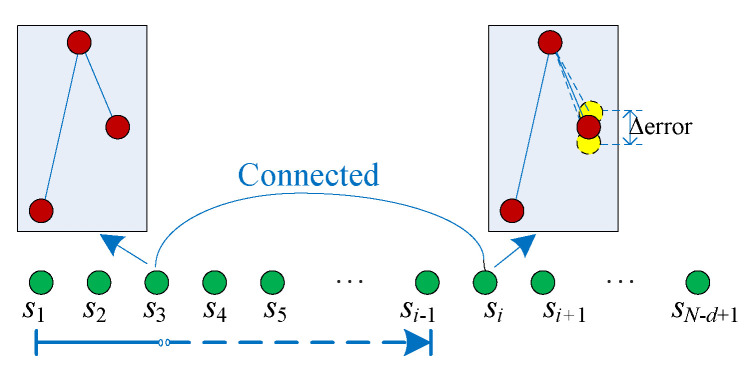
Building the network using the patterns and their weights.

The schematic diagram of the NPE algorithm is presented in Figure 2. Meanwhile, a toy example is given to help describe the procedure. Let us take the time series {x(n): 1 3 4 5 4 5 3 1 2 4 5 3} as the example. When d=3, the vectors are defined as {X(n)=[x(n,n+1,n+2)]},n=1,2,⋯,10. The Bandt–Pompe pattern series is {s(n): 123, 123, 132, 213, 312, 321, 231, 123, 123, 312}, and the weights are {w(n): 1.5556, 0.6667, 0.2222, −0.2222, 0.6667, 2.6667, −0.6667, −1.5556, 1.5556, 0.6667}. Here, set error=0.5. Now, we need to scan the pattern series and weight series. Let us start at i=2. We need to check whether s(1)=s(2). Since s(1)=s(2) and $\left|w\left(1\right)-w\left(2\right)\right|=0.8889>error$, the connection is not found. Increase the value of *i*, Let it be 3–7, and check whether the connections can be found between s(i) and s(1→i−1). There are no connections found since s(i)≠s(1→i−1)(i=3,⋯7). When i=8 and 9, there exists s(8,9)=s(1), but only $\left|w\left(9\right)-w\left(1\right)\right|<error$. Until now, the first connection is found between vector X(1) and X(9). When i=10, we have s(10)=s(5), and $\left|w\left(5\right)-w\left(10\right)\right|=0<error$. Thus, the second connection is found between vectors X(10) and X(5).

Firstly, the NPE is proposed based on the Bandt–Pompe patterns and their weights. Thus, it contains the characteristics of PE algorithm and WPE algorithm. Secondly, the connections are checked to build the probability distribution. In this method, the smaller the error is, the more difficult it is to find a connection. Thirdly, based on the matrix *M*, the corresponding network can be plotted to show the complexity of the time series. Finally, to better show the process of the NPE, the pseudocode of NPE is illustrated in Algorithm 2.

**Algorithm 2** Pseudocode of the NPE algorithm.
**Input:** *x*, *d*, error**Output:** NPE, *P*, *M* 
N=length(x);
 M=zeros(N−d+1,N−d+1); s=zeros(1,N−d+1); w=zeros(1,N−d+1); $P=10^{-5}\times ones\left(1,N-d+1\right)$; 
**for** i=1→N−d+1 **do**
  X=x(i:i+d−1);  s(i)=GetPattern(X);  $flag_{i}=mean\left(X\left(2:d-1\right)\right)-mean\left(X\right)$;  
**if** $flag_{i}\geq 0$ **then**
    $w(i)=\frac{1}{d}\sum_{k=1}^{d}{\left[x\left(i+k-1\right)-\bar{X}\left(i\right)\right]}^{2}$;  
**else**
    $w(i)=-\frac{1}{d}\sum_{k=1}^{d}{\left[x\left(i+k-1\right)-\bar{X}\left(i\right)\right]}^{2}$;  
**end if**
 
**end for**
 
**for** i=2→N−d+1 **do**
  
$Pat_{1}=s\left(i\right);$
  
$Wet_{1}=w\left(i\right);$
  
**for** j=1→i−1 **do**
    
$Pat_{2}=s\left(j\right);$
    
$Wet_{2}=w\left(j\right);$
    **if**$Pat_{1}==Pat_{2}$ & $\left|Wet_{1}-Wet_{2}\right|\leq error$ **then**      
$\left\{\begin{array}{c} P\left(j\right)=P\left(j\right)+1M\left(i,j\right)=1M\left(j,i\right)=1 \end{array}\right.$
      **Break**;    
**end if**
  
**end for**
 
**end for**
 
P=P/sum(P);
 
$NPE\left(x^{N},d,error\right)=-\frac{1}{\log\left(N-d+1\right)}\sum_{i=1}^{N-d+1}P\left(i\right)\log\left(P\left(i\right)\right);$



### 2.4. Performance of the Algorithm

To analyze the proposed NPE algorithm, four segments of time series with a length of $10^{3}$ are generated. Firstly, a random time series and a periodic time series, which is denoted as {1,2,3,4,1,2,3,4,1,2,3,4,⋯}, are generated. Then, two chaotic time series are generated. One is produced by the logistic map:

(14) $$x\left(n+1\right)=\mu x\left(n\right)\left(1-x\left(n\right)\right),$$

where μ=4; the other one is produced by the simplified Lorenz system [54]:

(15) $$\left\{\begin{array}{c} {\dot{x}}_{1}=10\left(x_{2}-x_{1}\right)\\ {\dot{x}}_{2}=\left(24-4c\right)x_{1}-x_{1}x_{3}+cx_{2}\\ {\dot{x}}_{3}=x_{1}x_{2}-8x_{3}/3 \end{array}\right.,$$

where the bifurcation parameter is c=2.

**Figure 3:**
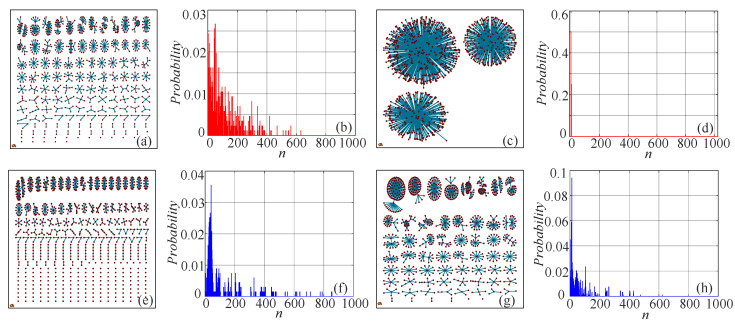
Networks and the corresponding probability distributions of different signals. (**a**) The network of the random signal; (**b**) the probability distribution of the random signal; (**c**) the network of the periodic signal; (**d**) the probability distribution of the periodic signal; (**e**) the network of the simplified Lorenz system with c=2; (**f**) the probability distribution of the simplified Lorenz system with c=2; (**g**) the network of the logistic map with μ=4; (**h**) the probability distribution of the logistic map with μ=4.

The networks and probability distributions of different kinds of time series are shown in Figure 3, where d=3, error=0.005. Figure 3a,b shows the network and probability distribution of a random time series. Compared with other cases, the random sequence has a more uniform probability distribution. Specifically, the periodic signal has a concentrated distribution. For many complexity measuring algorithms, including PE, ApEn, SampEn, and FuzzyEn, the measuring results for discrete chaotic systems are significantly higher than those of continuous chaotic systems. However, as shown in Figure 3, the uniformity of the the two probability distributions is at about the same level, which means that the measuring results of continuous chaotic systems and discrete chaotic systems could be at about the same level. Moreover, as shown in those networks, the more complex the time series has, the more different the structures presented.

**Figure 4:**
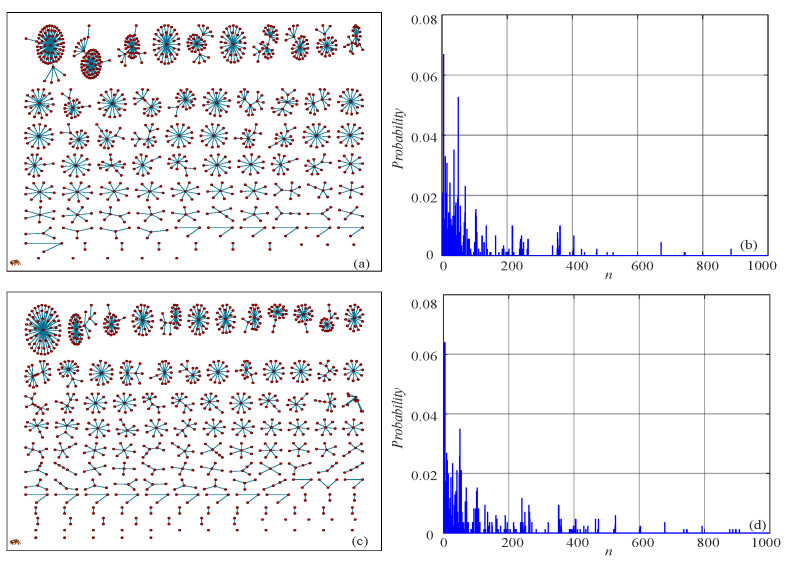
Networks and the corresponding probability distributions of the logistic map with different *d*. (**a**) The network with d=4; (**b**) the probability distribution with d=4; (**c**) the network with d=5; (**d**) the probability distribution with d=5.

**Figure 5:**
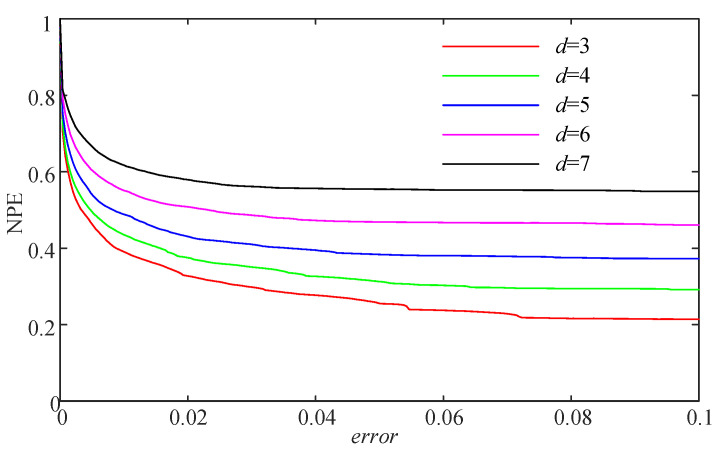
NPE measuring results versus the parameter error and different *d*.

Let d=4 and d=5; the networks and the corresponding probability distributions of the logistic map are illustrated in Figure 4. Basically, the different values of *d* can deduce the complexity of the time series. Meanwhile, the NPE of the logistic map time series with increasing error and different *d* is shown in Figure 5. It is shown in Figure 5 that the NPE decreases with the increase of error at the beginning, then it remains stable. According to the NPE algorithm, a smaller error means more difficulty in finding a connection. As a result, the probability distribution *P* is more “flat”, and the estimated NPE value is larger. Meanwhile, it is shown in Figure 5 that the NPE has a larger measuring result with larger *d*. In this paper, we chose d>3 for real applications.

## 3. Applications to Chaotic Systems

Chaotic systems can be divided into continuous chaotic systems and discrete chaotic systems. Furthermore, those systems can be classified into fractional-order chaotic systems and integer-order chaotic systems. Meanwhile, the chaotic systems have rich dynamics with the variation of the system parameters. NPE is applied to analyze the complexity of different chaotic systems.

### 3.1. Applications to the Integer-Order Chaotic Systems

**Figure 6:**
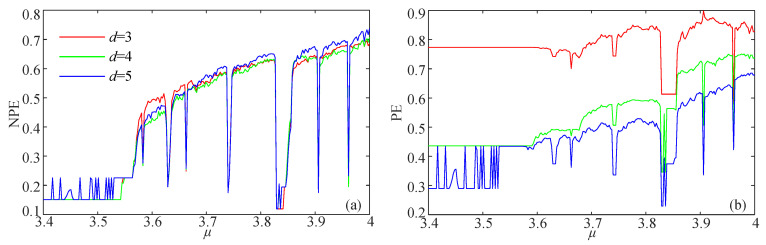
Complexity analysis results of the logistic map with the parameter μ varying. (**a**) NPE; (**b**) PE.

**Figure 7:**
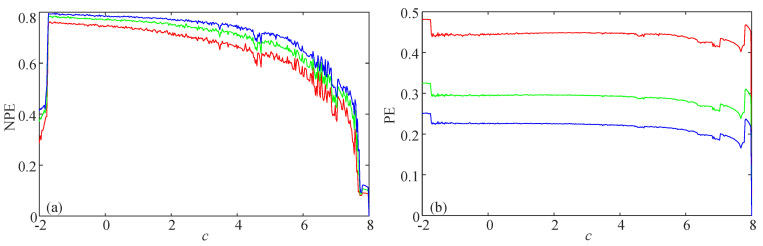
Complexity analysis results of the simplified Lorenz system with the parameter *c* varying. (**a**) NPE; (**b**) PE.

**Figure 8:**
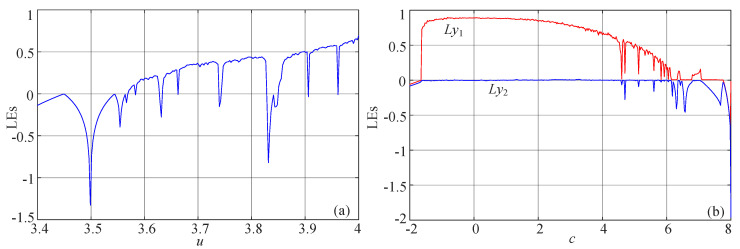
LEs of the chaotic systems. (**a**) Logistic map with the parameter μ varying; (**b**) simplified Lorenz system with parameter *c* varying.

Here, the complexity of the logistic map and the simplified Lorenz system are analyzed. The equations of these two systems are presented in Equations (14) and (15), respectively. The analysis results for the logistic map are shown in Figure 6. The parameter μ varies from 3.4–4 with a step size of Δμ=0.0024. Meanwhile, the NPE and PE complexity analysis results of the simplified Lorenz system are illustrated in Figure 7, where the bifurcation parameter varies from −2–8 with a step size of Δc=0.04. It is shown in Figure 7 that the complexity of the simplified Lorenz system decreases with the increase of *c*. Compared with the PE measuring result, the NPE measuring result has better consistency with the corresponding Lyapunov exponents, which are shown in Figure 8a. In fact, the maximum Lyapunov exponents (MLEs) of the simplified Lorenz system are shown in Figure 8b. It is shown that the NPE analysis results agree better with the MLEs of the system when compared with the PE results. For the PE algorithm, when the system is periodic, the measuring results can be larger than those of the chaotic state. As with the PE algorithm, the NPE algorithm is reliable for complexity analysis of chaotic systems.

### 3.2. Applications to the Fractional-Order Chaotic Systems

The fractional-order Hénon map is defined as:

(16) $$\left\{\begin{array}{c} {}^{C}\Delta_{t_{0}}^{\nu }x\left(i\right)=1-ax^{2}\left(i+v-1\right)+y\left(i+v-1\right)\\ \;\;\;\;\;\;-x\left(i+v-1\right)\\ {}^{C}\Delta_{t_{0}}^{\nu }y\left(i\right)=bx\left(i+v-1\right)-y\left(i+v-1\right) \end{array}\right.,$$

where $t\in N_{t_{0}+1-v},\;\;\;\;\;0<v\leq 1$ and *a* and *b* are the bifurcation parameters. Here, the Caputo difference ${}^{C}\Delta_{t_{0}}^{\nu }x$ [55] is used. By employing the numerical solution scheme, its solution is denoted as:

(17) $$\left\{\begin{array}{c} x\left(i\right)=x\left(0\right)+\sum_{j=1}^{n}\varphi \left(i,j,\upsilon \right)\left[1-ax^{2}\left(j-1\right)+y\left(j-1\right)-x\left(j-1\right)\right]\\ y\left(i\right)=y\left(0\right)+\sum_{j=1}^{n}\varphi \left(i,j,\upsilon \right)\left[bx\left(j-1\right)-y\left(j-1\right)\right] \end{array}\right.,$$

where $\varphi \left(i,j,\upsilon \right)=\frac{\Gamma \left(i-j+\nu \right)}{\Gamma \left(\nu \right)\cdot \Gamma \left(i-j+\nu \right)}$.

**Figure 9:**
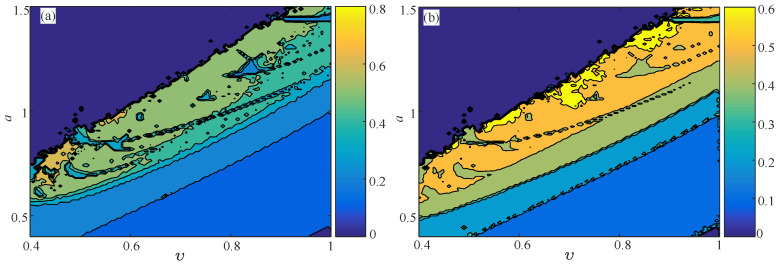
Complexity analysis results of the fractional-order Hénon map. (**a**) NPE; (**b**) PE.

**Figure 10:**
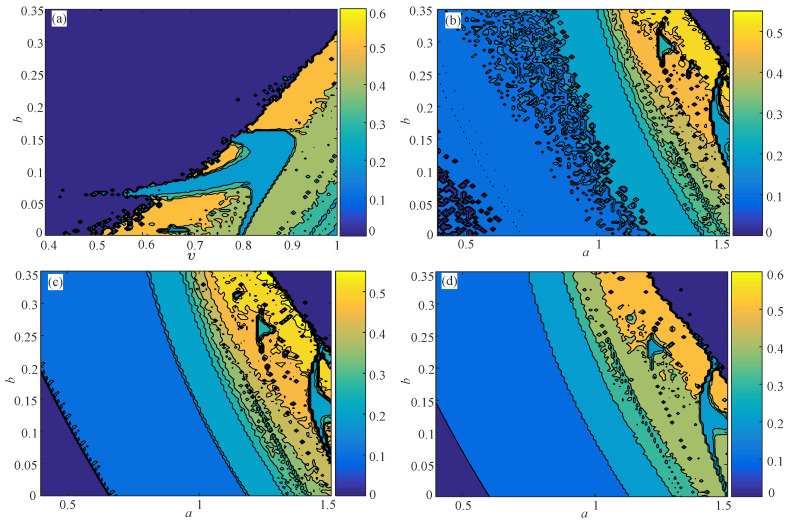
NPE complexity analysis results of the fractional-order Hénon map in different parameter planes (**a**) Parameter plane b−υ; (**b**) parameter plane a−b with υ=1; (**c**) parameter plane a−b with υ=0.95; (**d**) parameter plane a−b with υ=0.9.

The complexity of the fractional-order Hénon map is analyzed in the parameter planes b−ν, a−ν, and a−b. The parameter *a* varies from 0.4–1.5 with a step size of Δa=0.011; the parameter *b* varies from 0–0.35 with a step size of Δb=0.0035; and the fractional derivative order ν varies from 0.4–1 with a step size of Δν=0.006. Thus, each parameter plane is divided as a 101×101 grid. By fixing b=0.2, d=5, Figure 9 shows the complexity analysis results in the a−ν parameter plane. It is shown that both the NPE and PE algorithms are effective for the complexity analysis of the fractional-order Hénon map, since both the NPE and PE analysis results agree well with the SALIdetecting result and the SE analysis result as given in [56]. Moreover, the NPE in the parameter planes b−ν and a−b is shown in Figure 10. Here, the complexity in the a−b parameter plane is analyzed with different ν. According to Figure 10, the fractional-order Hénon map has a wide region of high complexity, and it provides a good model for real applications.

The fractional-order simplified Lorenz system is given by [56]:

(18) $$\left\{\begin{array}{c} D_{t_{0}}^{q}x_{1}=10\left(x_{2}-x_{1}\right)\\ D_{t_{0}}^{q}x_{2}=\left(24-4c\right)x_{1}-x_{1}x_{3}+cx_{2},\\ D_{t_{0}}^{q}x_{3}=x_{1}x_{2}-8x_{3}/3 \end{array}\right.$$

where $x_{1}$, $x_{2}$, and $x_{3}$ are state variables. *c* is the bifurcation parameter, and *q* is the fractional derivative order. In this study, the Caputo fractional calculus [57] is used, and the system is solved by the Adams–Bashforth–Moulton (ABM) algorithm [58].

**Figure 11:**
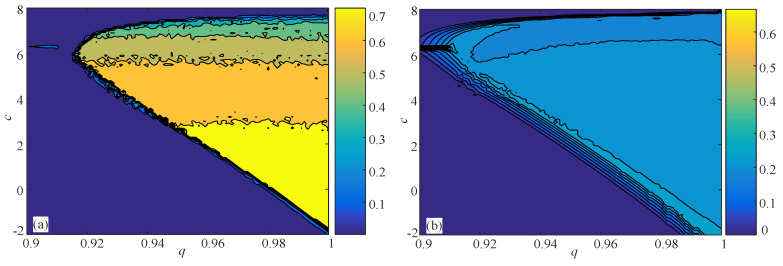
Complexity analysis results of the fractional-order simplified Lorenz system. (**a**) NPE; (**b**) PE.

Let the derivative order *q* vary from 0.9–1 with a step size of Δq=0.004 and the bifurcation parameter *c* vary from −2–8 with a step size of Δc=0.04. The analysis results are shown in Figure 11. The high complexity region matches well with the results in the previous work [56]. It shows the effectiveness of the proposed NPE algorithm. Meanwhile, it is shown in Figure 11 that the NPE produces good measure results as the PE algorithm analysis results for the continuous chaotic system.

## 4. Applications to EEG Signals

The EEG signals of three groups [52], which are denoted as A001–A100, C001–C100, and E001–E100, were analyzed. The descriptions of each group of EEG recordings are presented in Table 1. The data were downloaded from the web site http://epileptologie-bonn.de/cms/front_content.php?idcat=193&lang=3&changelang=3. According to [52], the chosen Dataset A is the surface EEG recordings that were obtained from five healthy volunteers using a standardized electrode placement scheme, while Datasets C and E originated from the EEG archive of presurgical diagnosis.

**Figure 12:**
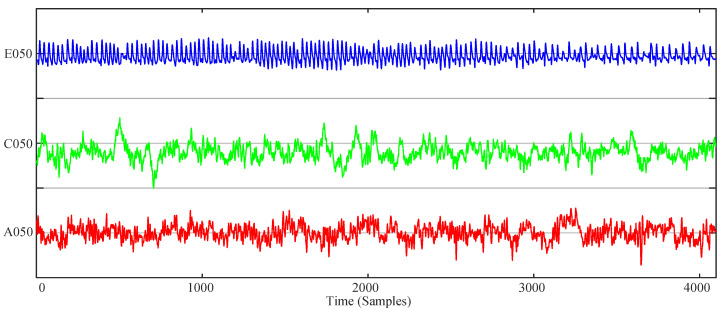
Sample data of A050, C050, and E050.

Specifically, segments of Group A were taken from the depicted electrodes of healthy volunteers, Segments of Group C were taken from the depicted electrodes of the epileptogenic zone of patients during the seizure-free intervals, and segments of Group E were taken from the depicted electrodes of epileptogenic zone of patients during seizure activity. Figure 12 shows the data of A050, C050, and E050. More details about the EEG signals can be found in [11,29,52].

We investigated the complexity of these EEG signals by employing the fractional FuzzyEn algorithm [11] and the multiscale permutation Rényi entropy [29]. The effectiveness of the proposed methods is shown. In fact, the NPE algorithm can be used to analyze the complexity of different kinds of time series including these biomedical signals.

In this study, the NPE was employed to measure the complexity of the EEG signals and to verify the effectiveness of the proposed method. As in [11,29], the sliding window method was employed, where the length of each window was 1000, the sliding step was 30, and the number of windows was 100.

**Figure 13:**
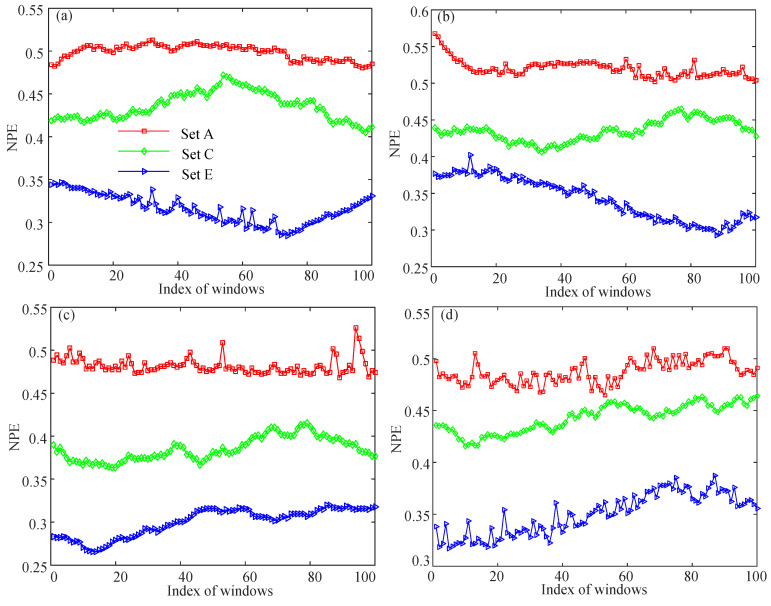
NPE complexity analysis results of four data segments. (**a**) A020, C020 and E020; (**b**) A040, C040 and E040; (**c**) A070, C070 and E070; (**d**) A100, C100 and E100.

Firstly, the NPE complexity of (A020, C020, E020), (A040, C040, E040), (A070, C070, E070), and (A100, C100, E100) was analyzed, and the results are shown in Figure 13. It is shown that the NPE algorithm can distinguish different states. As shown in Figure 13, the EEG signal of the epileptic patients during seizure activity had the lowest complexity, while the EEG signal of healthy volunteers had the largest complexity. According to [11,29], the EEG and ECG signals of healthy persons had higher complexity than the signals of unhealthy persons. As shown in Figure 13, the epileptic patients during both the seizure-free intervals and the seizure activity intervals had EEG signals of lower complexity than those of the healthy volunteers.

**Figure 14:**
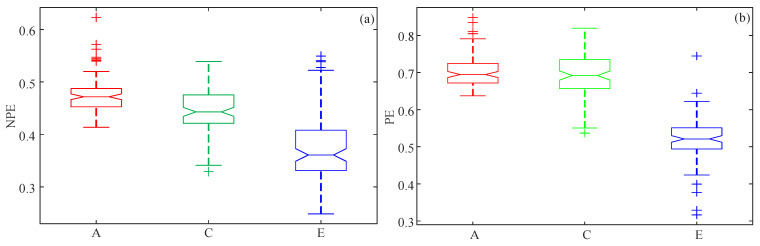
Boxplots of the complexity results for Set A, Set C, and Set E. (**a**) NPE; (**b**) PE.

Secondly, the PE and NPE complexity of all the data was estimated, and its statistic analysis was carried out. We took the mean value of the 100 measuring results as the final PE and NPE result of each signal. Then, the NPE results of A001–A100, C001–C100, and E001–E100 were obtained. The analysis results are illustrated in the boxplot in Figure 14.

Obviously, the NPE results showed that the rank of complexity yield was Set A > Set C > Set E. However, to verify this result, further statistical analysis was needed. In this study, one-way analysis of variance (ANOVA) was applied to check whether the deduced conclusion was true. Meanwhile, the experiments were carried out by employing MATLAB by using the function p=anova1(X), where *X* contains the NPE results. As a result, Figure 14a was produced by this function. Meanwhile, Table 2 shows that the *p*-value was $1.54997\times 10^{-35}$ and the *F* value was 106. Since the *p*-value was smaller than 0.005, we could reject the null hypothesis, which meant that the NPE complexity analysis results of Set A, Set C, and Set E were statistically significant. Table 3 illustrates the statistical analysis results between Set A and Set C, Set A and Set E, and Set C and Set E. It is shown in Table 3 that all *p*-values were smaller than 0.005; thus, the analysis results were statistically significant among each other. Figure 14b shows the boxplot of the PE algorithm-based complexity analysis results. It indicates that the PE complexity of Set A data and Set C data overlapped with each other greatly. According to Table 4, the *p*-value between Set A and Set C was 0.1682, which is larger than 0.005. This meant that there was no difference between the PE measure results for Set A data and Set C data, statistically. Thus, the PE algorithm cannot distinguish Set A and Set C. In conclusion, the NPE algorithm was an effective method for the complexity of nonlinear time series, and the complexity of the three sets of EEG signals were ranked as Set A > Set C > Set E.

## 5. Discussions and Conclusions

Currently, many entropy measure algorithms have been proposed where the Shannon entropy or generalized fractional entropy was employed. Thus, how to extract a proper probability distribution that can reflect more information of the time series is the key for a more satisfying entropy result. In this paper, the network permutation entropy (NPE) was proposed to measure the complexity of nonlinear time series. Compared to the Bandt–Pompe probability distribution in the PE algorithm, the probability distribution of the NPE algorithm considered both the Bandt–Pompe patterns and the weights of the reconstructed vectors. Moreover, let us take the given periodic time series {1, 2, 3, 4, 5, 1, 2, 3, 4, 5, 1, 2, ...} of length 20,480 as an example to show the superiority of the NPE algorithm for the periodic time series. The estimated value for the normalized PE with d=3 is PE=0.5303, with d=4 is PE=0.4192, with d=5 is PE=0.3362, and with d=6 is PE=0.2446. The measure results of this periodic time series decrease with the increase of *d*. However, when the complexity of the time series is measured by the NPE with error=0.0005, the results are d=3 for NPE=0.0957, d=4 for NPE=0.1342, d=5 for NPE=0.1621, and d=6 for NPE=0.1621. Thus, the complexity measure results of the new method are much closer to the ideal minimum value (zero) for the complexity of “simple” time series. Since the NPE algorithm combines the characteristics and advantage of PE, WPE and the network and extracts more information about the nonlinearity of the time series, a more satisfying measure result is obtained. Meanwhile, there are many other network entropy algorithms proposed. Compared with those network entropy algorithms, the NPE algorithm had about the same the calculation complexity since the time and space complexity degrees were both defined as O(n).

As an application, NPE was employed to analyze the complexity of different chaotic systems including the logistic map, the integer-order simplified Lorenz system, the fractional-order Hénon map, and the fractional-order simplified Lorenz system. It was shown that the NPE was an effective method for complexity analysis of chaotic systems. Both the PE and NPE measured the complexity of chaotic systems effectively, but the analysis results of the NPE algorithm matched better with the MLEs of the continuous chaotic systems. Meanwhile, the NPE algorithm-based contour plots showed that the systems had wide ranges of high complexity. Those contour plots showed more information on the dynamics of the chaotic systems. This provides a new approach for the parameter choice of chaotic systems in real applications.

Moreover, the nature of the complexity of EEG signals was investigated by employing the NPE algorithm. Here, the EEG signals were exemplary time series such as Z093, O015, N062, F021, and S056. The statistic analysis between the complexity analysis results of different sets of data was carried out based on the ANOVA and LSD. Accordingly, the NPE algorithm could distinguish different sets of EEG data. Meanwhile, the analysis results showed that the NPE complexity in descending order was: Set A (healthy volunteers), Set C (epileptic patients during seizure-free intervals), and Set E (epileptic patients during seizure activity). Hence, it was shown that the proposed NPE was effective for the complexity analysis of nonlinear time series. Our future work will focus on the complexity analysis of more real biological signals such as EEG signals and ECG signals by employing the proposed NPE algorithm and a proper neural network.

## Figures and Tables

**Table 1:** Descriptions of the chosen datasets.

Datasets	Data Description
Set A	Signals from healthy volunteers relaxed in an awake state with eyes open; data are presented as A001–A100
Set C	Signals from epileptic patients during seizure-free intervals; data are presented as C001–C100
Set E	Signals from epileptic patients during seizure activity; data are presented as E001–E100

**Table 2:** ANOVA table for the NPE analysis results.

Source	SS	df	MS	*F*	*p*-Value
Columns	0.52932	2	0.2646	106	$1.54997\times 10^{-35}$
Error	0.73972	297	0.00249	-	-
Total	1.26904	299	-	-	-

**Table 3:** Values of (p,F) of the LSDfor the NPE analysis results.

	A	C	E
A	-	($1.21401\times 10^{-7}$, 30.16)	($2.80255\times 10^{-30}$, 185.84)
C	($1.21401\times 10^{-7}$, 30.16)	-	($1.06364\times 10^{-15}$, 76.2)
E	($2.80255\times 10^{-30}$, 185.84)	($1.06364\times 10^{-15}$, 76.2)	-

**Table 4:** Values of (p,F) of the LSD for the PE analysis results.

	A	C	E
A	-	(0.1682, **1.91**)	($6.50147\times 10^{-64}$, 639)
C	(0.1682, **1.91**)	-	($2.28427\times 10^{-51}$, 427.8)
E	($6.50147\times 10^{-64}$, 639)	($2.28427\times 10^{-51}$, 427.8)	-

## Abbreviations

The following abbreviations are used in this manuscript:

PE	Permutation entropy
NPE	Network permutation entropy

## References

[1] Ocak H. (2009). Automatic detection of epileptic seizures in EEG using discrete wavelet transform and approximate entropy. Expert Syst. Appl..

[2] Li J., Yan J., Liu X., Ouyang G. (2014). Using permutation entropy to measure the changes in EEG signals during absence seizures. Entropy.

[3] Li T., Zhou M. (2016). ECG classification using wavelet packet entropy and random forests. Entropy.

[4] Kumar M., Pachori R.B., Acharya U.R. (2018). Automated diagnosis of atrial fibrillation ECG signals using entropy features extracted from flexible analytic wavelet transform. Biocybern. Biomed. Eng..

[5] Dostál O., Vysata O., Pazdera L., Procházka A., Kopal J., Kuchyňka J., Vališ M. (2018). Permutation entropy and signal energy increase the accuracy of neuropathic change detection in needle EMG. Comput. Intell. Neurosci..

[6] Jiang L., Wu K., Zhou G. (2018). Asymmetry in stock comovements: An entropy approach. J. Financ. Quant. Anal..

[7] Zhang Y., Shang P. (2019). The complexity–entropy causality plane based on multivariate multiscale distribution entropy of traffic time series. Nonlinear Dyn..

[8] Zhang Y., Yang X., Cattani C. (2016). Tea category identification using a novel fractional fourier entropy and jaya algorithm. Entropy.

[9] He S., Li C., Sun K., Jafari S. (2018). Multivariate multiscale complexity analysis of self-reproducing chaotic systems. Entropy.

[10] Natiq H., Said M., Al-Saidi N., Kilicman A. (2019). Dynamics and complexity of a new 4d chaotic laser system. Entropy.

[11] He S., Sun K., Wang R. (2018). Fractional fuzzy entropy algorithm and the complexity analysis for nonlinear time series. Eur. Phys. J. Spec. Top..

[12] Ran J., Li Y., Wang C. (2018). Chaos and complexity analysis of a discrete permanent-magnet synchronous motor system. Complexity.

[13] He S., Sun K., Wang H. (2015). Complexity analysis and DSP implementation of the fractional-order Lorenz hyperchaotic system. Entropy.

[14] Shannon C.E. (1948). A mathematical theory of communication. Bell Syst. Tech. J..

[15] Kolmogorov A.N. (1965). Three approaches to the definition of the concept “quantity of information”. Probl. Peredachi Inf..

[16] Li M., Vitányi P. (2013). An Introduction to Kolmogorov Complexity and Its Applications.

[17] Cai Z., Sun J. (2009). Convergence of C_0_ complexity. Int. J. Bifurc. Chaos.

[18] Pincus S. (1995). Approximate entropy (ApEn) as a complexity measure. Chaos Interdiscip. J. Nonlinear Sci..

[19] Richman J.S., Moorman J.R. (2000). Physiological time-series analysis using approximate entropy and sample entropy. Am. J. Physiol. Heart Circ. Physiol..

[20] Chen W., Zhuang J., Yu W., Wang Z. (2009). Measuring complexity using FuzzyEn, ApEn, and SampEn. Med Eng. Phys..

[21] Lempel A., Ziv J. (1976). On the complexity of finite sequences. IEEE Trans. Inf. Theory.

[22] Lopez-Ruiz R., Mancini H.L., Calbet X. (1995). A statistical measure of complexity. Phys. Lett. A.

[23] Bandt C., Pompe B. (2002). Permutation entropy: A natural complexity measure for time series. Phys. Rev. Lett..

[24] Staniczenko P.P., Lee C.F., Jones N.S. (2009). Rapidly detecting disorder in rhythmic biological signals: A spectral entropy measure to identify cardiac arrhythmias. Phys. Rev. E.

[25] Rosso O.A., Blanco S., Yordanova J., Kolev V., Figliola A., Schürmann M., Başar E. (2001). Wavelet entropy: A new tool for analysis of short duration brain electrical signals. J. Neurosci. Methods.

[26] Costa M., Goldberger A.L., Peng C.K. (2005). Multiscale entropy analysis of biological signals. Phys. Rev. E.

[27] Humeau-Heurtier A., Wu C.W., Wu S.D. (2015). Refined composite multiscale permutation entropy to overcome multiscale permutation entropy length dependence. IEEE Signal Process. Lett..

[28] Wu S.D., Wu C.W., Lee K.Y., Lin S.G. (2013). Modified multiscale entropy for short-term time series analysis. Phys. A Stat. Mech. Appl..

[29] Yin Y., Sun K., He S. (2018). Multiscale permutation Rényi entropy and its application for EEG signals. PLoS ONE.

[30] Labate D., La Foresta F., Morabito G., Palamara I., Morabito F.C. (2013). Entropic measures of EEG complexity in Alzheimer’s disease through a multivariate multiscale approach. IEEE Sens. J..

[31] Zunino L., Olivares F., Scholkmann F., Rosso O.A. (2017). Permutation entropy based time series analysis: Equalities in the input signal can lead to false conclusions. Phys. Lett. A.

[32] Bian C., Qin C., Ma Q.D., Shen Q. (2012). Modified permutation-entropy analysis of heartbeat dynamics. Phys. Rev. E.

[33] Fadlallah B., Chen B., Keil A., Príncipe J. (2013). Weighted-permutation entropy: A complexity measure for time series incorporating amplitude information. Phys. Rev. E.

[34] Azami H., Escudero J. (2016). Amplitude-aware permutation entropy: Illustration in spike detection and signal segmentation. Comput. Methods Programs Biomed..

[35] Chen Z., Li Y., Liang H., Yu J. (2019). Improved permutation entropy for measuring complexity of time series under noisy condition. Complexity.

[36] Gao Z.K., Cai Q., Yang Y.X., Dang W.D., Zhang S.S. (2016). Multiscale limited penetrable horizontal visibility graph for analyzing nonlinear time series. Sci. Rep..

[37] Zou Y., Donner R.V., Marwan N., Donges J.F., Kurths J. (2018). Complex network approaches to nonlinear time series analysis. Phys. Rep..

[38] Zhang H., Meng Q., Liu M., Li Y. A new epileptic seizure detection method based on fusion feature of weighted complex network. Proceedings of the International Symposium on Neural Networks.

[39] Wang C., Zhang Z., Zhu M. Nonlinear dynamic analysis of air traffic flow at different temporal scales: Nonlinear Analysis approach versus complex networks approach. Proceedings of the 2018 IEEE International Conference on Software Quality, Reliability and Security Companion (QRS-C).

[40] Xie W.J., Han R.Q., Zhou W.X. (2019). Tetradic motif profiles of horizontal visibility graphs. Commun. Nonlinear Sci. Numer. Simul..

[41] Gao Z., Wang X., Yang Y. (2019). EEG-Based Spatio-Temporal Convolutional Neural Network for Driver Fatigue Evaluation. IEEE Trans. Neural Netw. Learn. Syst..

[42] Chai R., Naik G.R., Nguyen T.N., Ling S.H., Tran Y., Craig A., Nguyen H.T. (2016). Driver fatigue classification with independent component by entropy rate bound minimization analysis in an EEG-based system. IEEE J. Biomed. Health Inform..

[43] Liu G., Zhang Y., Hu Z., Du X., Wu W., Xu C., Wang X., Li S. (2017). Complexity analysis of electroencephalogram dynamics in patients with Parkinson’s disease. Parkinson’s Dis..

[44] Al-Ani A., Koprinska I., Naik G. (2017). Dynamically identifying relevant EEG channels by utilizing channels classification behaviour. Expert Syst. Appl..

[45] Stokić M., Milovanović D., Ljubisavljević M.R., Nenadović V., Čukić M. (2015). Memory load effect in auditory–verbal short-term memory task: EEG fractal and spectral analysis. Exp. Brain Res..

[46] Butkevičiūtė E., Bikulčienė L., Sidekerskienė T., Blažauskas T., Maskeliūnas R., Damaševičius R., Wei W. (2019). Removal of movement artefact for mobile EEG analysis in sports exercises. IEEE Access.

[47] Nejedly P., Cimbalnik J., Klimes P., Plesinger F., Halamek J., Kremen V., Viscor I., Brinkmann B.H., Pail M., Brazdil M. (2019). Intracerebral EEG artifact identification using convolutional neural networks. Neuroinformatics.

[48] Kilicarslan A., Contreras-Vidal J.L. (2019). Characterization and real-time removal of motion artifacts from EEG signals. J. Neural Eng..

[49] Acharyya A., Jadhav P.N., Bono V., Maharatna K., Naik G.R. (2018). Low-complexity hardware design methodology for reliable and automated removal of ocular and muscular artifact from EEG. Comput. Methods Programs Biomed..

[50] Bhardwaj S., Jadhav P., Adapa B., Acharyya A., Naik G.R. Online and automated reliable system design to remove blink and muscle artefact in EEG. Proceedings of the 2015 37th Annual International Conference of the IEEE Engineering in Medicine and Biology Society (EMBC).

[51] Jadhav P., Shanamugan D., Chourasia A., Ghole A., Acharyya A., Naik G. Automated detection and correction of eye blink and muscular artifacts in EEG signal for analysis of Autism Spectrum Disorder. Proceedings of the 2014 36th Annual International Conference of the IEEE Engineering in Medicine and Biology Society.

[52] Andrzejak R.G., Lehnertz K., Mormann F. (2001). Indications of nonlinear deterministic and finite-dimensional structures in time series of brain electrical activity: Dependence on recording region and brain state. Phys. Rev. E.

[53] He S., Sun K., Wang H. (2016). Multivariate permutation entropy and its application for complexity analysis of chaotic systems. Physica A.

[54] Sun K., Sprott J.C. (2009). Dynamics of a simplified Lorenz system. Int. J. Bifurc. Chaos.

[55] Khennaoui A.A., Ouannas A., Bendoukha S., Grassi G., Lozi R.P., Pham V.T. (2019). On fractional–order discrete–time systems: Chaos, stabilization and synchronization. Chaos Solitons Fractals.

[56] He S., Sun K., Peng Y. (2019). Detecting chaos in fractional-order nonlinear systems using the smaller alignment index. Phys. Lett. A.

[57] Gorenflo R., Mainardi F. (1997). Fractional calculus. Fractals and Fractional Calculus in Continuum Mechanics.

[58] Diethelm K. (1997). An algorithm for the numerical solution of differential equations of fractional order. Electron. Trans. Numer. Anal.

